# Supplementary material for: Mitogenomic Insights into the Evolution, Divergence Time, and Ancestral Ranges of Coturnix Quails
Source: Genes (Basel). 2024 Jun 5;15(6):742. doi: 10.3390/genes15060742 (PMC11202683; doi:10.3390/genes15060742)
Supplement: Supplementary file 1 [file genes-15-00742-s001.zip › Supplementary_Figure_legend.pdf]

Supplementary Figure Legend

Figure S1. Secondary structures of the 22 tRNA genes for the *Coturnix japonica* mitogenome.

Figure S2. Secondary structures of the 22 tRNA genes for the *Coturnix coturnix* mitogenome.

Figure S3. Graphical representation of (A) Codon usage analysis and (B) Relative synonymous codon usage (RSCU) analysis for the *Coturnix japonica* (blue) and *Coturnix coturnix* (orange) mitogenomes sequenced in this study. Most prevalent codons for each amino acid are also indicated in the figure. Codon usage and RSCU analysis revealed identical preferences in amino acid and codon usages.

Figure S4. The 13 protein coding genes of select species of the *Coturnix* lineage used in this study translated into respective amino acid sequences and aligned to study visual cues. The instances of non-synonymous substitutions in the *nad4* amino acid sequence alignment are highlighted in the blue dotted box. Various parameters of amino acid alignment are mentioned below each alignment. Visual inspection of the *nad4* amino acid sequence alignment shows 44 non-synonymous substitutions within the *Coturnix* lineage and the most frequent being were alanine to threonine (n=5).

Figure S5. Posterior parameters estimated from the Bayesian Inference (BI) tree constructed in this study. The ESS values of each chain, marginal density, and trace of runs was captured as a screenshot.

Figure S6. Posterior parameters estimated from the BEAST v2.6.7 run undertaken in this study. The ESS values of the run, marginal density and trace of run was captured as a screenshot.

Figure S7. Geographic range of *Coturnix coturnix* and *Coturnix japonica* showing extant and introduced areas of the respective species. Range of *Coturnix coturnix* is highlighted in yellow

and *Coturnix japonica* in orange. The Map was adapted with permission from the Birdlife International and Handbook of the Birds of the World (2017).

Figure S8. Maximum likelihood tree of 79 mitogenomes used for divergence time estimates in this study. The tree is constructed using IQTREE v1.6.12 and the placement of fossils (as in Supplementary Table 6) was indicated by A, B, C and D on the tree.

Figure S9. Heatmap of sequence divergence estimates among selected mitogenomes used in this study. The heatmap displays the estimated sequence divergence among 71 mitogenomes. Each cell in the heatmap represents the divergence score between pairs of sequences, with the colour intensity indicating the degree of divergence according to the scale shown on the right. Sequences are clustered based on their phylogenetic relationships, as indicated by the dendrogram on the top and/or side of the heatmap.

Figure S10. Heatmap of sequence divergence estimates among selected genera used in this study. This heatmap displays the estimated sequence divergence among 31 genera. Each cell in the heatmap represents the divergence score between pairs of genera, with the colour intensity indicating the degree of divergence according to the scale shown on the right. Sequences are clustered based on their phylogenetic relationships, as indicated by the dendrogram on the top and/or side of the heatmap.

Figure S11. Modified ancestral biogeographic area reconstruction estimated using the DEC+J model generated in BioGeoBEARS. BEAST maximum clade credibility tree was pruned to contain seven *Coturnix* and one *Alectoris* species (as out-group). The estimated ancestral areas are coloured according to the four zoogeographic zones as: (i) A (Oceanian and Australian), (ii) B (Oriental), (iii) western Palaearctic (C), (iv) eastern Palearctic and Sino-Japanese (E) and (v) D (Saharo-Arabian, Afro-Tropical and Madagascan). The colour palate on the nodes of the chronogram shows the combination of most likely ancestral area estimated by BioGeoBEARS.

Coding scheme for each geographic area for each species as present or absent is shown on the right of the tree. Insert: biogeographic areas used in the ancestral area reconstruction.
