# Supplementary material for: Mitogenomic Insights into the Evolution, Divergence Time, and Ancestral Ranges of Coturnix Quails
Source: Genes (Basel). 2024 Jun 5;15(6):742. doi: 10.3390/genes15060742 (PMC11202683; doi:10.3390/genes15060742)
Supplement: Supplementary file 1 [file genes-15-00742-s001.zip › Table S1.pdf]

Table S1. List of all mitogenomes downloaded from NCBI and used for investigations in this study.

S. No.	Species	NCBI accession no.
1	*Acryllium vulturinum*	NC014180
2	*Alectoris chukar*	NC020585
3	*Arborophila ardens*	NC022683
4	*Arborophila brunneopectus*	NC022684
5	*Arborophila rufipectus*	NC012453
6	*Arborophila rufogularis*	NC020584
7	*Bambusicola fytchii*	NC020583
8	*Bambusicola thoracica*	NC011816
9	*Caloperdix oculeus*	NC024619
10	*Chrysolophus amherstiae*	NC020590
11	*Chrysolophus pictus*	NC014576
12	*Columba livia*	NC013978.1
13	*Coturnix chinensis*	NC004575
14	*Coturnix coturnix*	MW574359
15	*Coturnix delegorguei*	MW574360
16	*Coturnix japonica*	KXY712089
17	*Coturnix japonica*	MW574361
18	*Coturnix japonica*	NC003408
19	*Coturnix pectoralis*	MW574362
20	*Coturnix ypsilophora*	MW574363
21	*Crossoptilon auritum*	NC015897
22	*Crossoptilon crossoptilon*	NC016679
23	*Crossoptilon harmani*	NC026547.1
24	*Crossoptilon mantchuricum*	NC026548.1
25	*Francolinus pintadeanus*	NC011817
26	*Gallus gallus*	NC053523
27	*Gallus lafayetii*	NC007239
28	*Gallus sonneratii*	NC007240
29	*Gallus varius*	NC007238
30	*Haematortyx sanguiniceps*	NC034001
31	*Ithaginis cruentus*	NC018033
32	*Lagopus lagopus*	NC035568
33	*Lagopus muta*	NC034002
34	*Lophophorus impejanus*	NC040850.1
35	*Lophophorus lhuysii*	NC013979
36	*Lophophorus sclateri*	NC020589
37	*Lophura ignita*	NC010781
38	*Lophura nycthemera*	NC012895
39	*Lophura swinhoii*	NC023779

40	*Lyrurus tetrix*	NC024554
41	*Margaroperdix madagarensis*	MW574377
42	*Meleagris gallopavo*	NC010195
43	*Pavo cristatus*	NC024533
44	*Pavo muticus*	NC012897
45	*Perdix dauurica*	NC020588
46	*Perdix hodgsoniae*	NC023940
47	*Perdix perdix*	NC039843
48	*Phasianus colchicus*	NC015526
49	*Phasianus versicolor*	NC010778
50	*Polyplectron bicalcaratum*	NC012900
51	*Polyplectron germaini*	NC023264
52	*Polyplectron malacense*	NC044743
53	*Polyplectron napoleonis*	NC024615
54	*Pternistis swainsonii*	MW574387
55	*Ptilopachus petrosus*	NC024616
56	*Pucrasia macrolopha*	NC020587
57	*Syrmaticus ellioti*	NC010771
58	*Syrmaticus humiae*	NC010774
59	*Syrmaticus reevesii*	NC010770
60	*Syrmaticus soemmerringi*	NC010767
61	*Tetrao parvirostris*	NC043949
62	*Tetraogallus himalayensis*	NC027279
63	*Tetraogallus tibetanus*	NC023939
64	*Tetraophasis obscurus*	NC018034
65	*Tetraophasis szechenyii*	NC020613
66	*Tetrastes bonasia*	NC020591
67	*Tetrastes sewerzowi*	NC025318
68	*Tragopan caboti*	NC013619
69	*Tragopan temminckii*	NC020586
	**Mitogenomes included only for divergence time estimation**	
70	*Anas penelope*	NC050973.1
71	*Anas platyrhynchos*	NC009684.1
72	*Callipepla squamata*	NC29340.1
73	*Colinus virginianus*	NC24620.1
74	*Tympanuchus cupido*	MW574394.1
75	*Leipoa ocellata*	MW574372.1
76	*Alectura lathami*	NC007227.1
77	*Odontophorus gujanensis*	NC52784.1
