# Supplementary material for: Mitogenomic Insights into the Evolution, Divergence Time, and Ancestral Ranges of Coturnix Quails
Source: Genes (Basel). 2024 Jun 5;15(6):742. doi: 10.3390/genes15060742 (PMC11202683; doi:10.3390/genes15060742)
Supplement: Supplementary file 1 [file genes-15-00742-s001.zip › Table S2.pdf]

Table S2. Fossils used for time-calibrating the Phasianidae phylogeny. Records are adapted from published Galliformes reports that best suited our dataset (Hosner et al. 2016, Wang et al. 2017, Chen et al. 2021).

	**Fossil record used**	**Locality**	**Node calibrated**	**Position on ML tree (Figure S1)**	**Hard minimum (Ma)**
1	*Gallinuloides wyomingensis*	Green River, Wyoming, USA	Galliformes + Anseriformes	A	51.6 Ma
2	*Schaubortyx keltica*	Armissan, France	Odontophoridae + Phasianidae	B	27.5 Ma
3	*Rhegminornis calobates*	Gilchrist Co., Florida, USA	Meleagris + Tympanuchus	C	18.0 Ma
4	*Progura gallinacea*	Darling Downs, Australia	Leipoa + Alectura	D	4.5 Ma
