# Supplementary material for: Mitogenomic Insights into the Evolution, Divergence Time, and Ancestral Ranges of Coturnix Quails
Source: Genes (Basel). 2024 Jun 5;15(6):742. doi: 10.3390/genes15060742 (PMC11202683; doi:10.3390/genes15060742)
Supplement: Supplementary file 1 [file genes-15-00742-s001.zip › Table S3.pdf]

Table S3. Structural features and annotation of *Coturnix coturnix* mitogenome sequenced in this study.

**Name**	**Start**	**Stop**	**Strand**	**Length**	**Start codon**	**Stop codon**
CR	1	1158				
trnF(gaa)	1159	1226	+	68		
rrnS	1226	2199	+	974		
trnV(tac)	2200	2270	+	71		
rrnL	2271	3883	+	1613		
trnL2(taa)	3884	3957	+	74		
nad1	3966	4940	+	975	ATG	TAA
trnI(gat)	4941	5010	+	70		
trnQ(ttg)	5016	5086	-	71		
trnM(cat)	5086	5154	+	69		
nad2	5155	6195	+	1041	ATG	TAG
trnW(tca)	6194	6269	+	76		
trnA(tgc)	6275	6343	-	76		
trnN(gtt)	6346	6418	-	69		
trnC(gca)	6419	6484	-	73		
trnY(gta)	6484	6554	-	66		
cox1	6556	8106	+	1551	GTG	AGG
trnS2(tga)	8098	8172	-	75		
trnD(gtc)	8175	8243	+	69		
cox2	8245	8928	+	684	ATG	TAA
trnK(ttt)	8930	8997	+	68		

atp8	8999	9166	+	168	ATG	TAA
atp6	9157	9840	+	684	ATG	TAA
cox3	9840	10623	+	784	ATG	T(AA)
trnG(tcc)	10624	10692	+	69		
nad3	10693	10865	+	352	ATG	TAA
	10867	11044	+			
trnR(tcg)	11046	11114	+	69		
nad4l	11115	11411	+	297	ATG	TAA
nad4	11405	12782	+	1378	ATG	T(AA)
trnH(gtg)	12783	12851	+	69		
trnS1(gct)	12852	12921	+	70		
trnL1(tag)	12922	12992	+	71		
nad5	12993	14813	+	1821	ATG	TAA
cob	14813	15955	+	1143	ATG	TAA
trnT(tgt)	15959	16028	+	70		
trnP(tgg)	16031	16100	-	70		
nad6	16106	16627	-	522	ATG	AGG
trnE(ttc)	16629	16696	-	68		
