# Supplementary material for: Mitogenomic Insights into the Evolution, Divergence Time, and Ancestral Ranges of Coturnix Quails
Source: Genes (Basel). 2024 Jun 5;15(6):742. doi: 10.3390/genes15060742 (PMC11202683; doi:10.3390/genes15060742)
Supplement: Supplementary file 1 [file genes-15-00742-s001.zip › Table S4.pdf]

Table S4. Structural features and annotation of *Coturnix japonica* mitogenome sequenced in this study.

**Name**	**Start**	**Stop**	**Strand**	**Length**	**Start codon**	**Stop codon**
CR	1	1155				
trnF(gaa)	1156	1224	+	68		
rrnS	1224	2197	+	974		
trnV(tac)	2198	2268	+	71		
rrnL	2269	3883	+	1615		
trnL2(taa)	3884	3957	+	74		
nad1	3966	4940	+	975	ATG	TAA
trnI(gat)	4941	5011	+	70		
trnQ(ttg)	5017	5087	-	71		
trnM(cat)	5087	5154	+	69		
nad2	5156	6196	+	1041	ATG	TAG
trnW(tca)	6195	6270	+	76		
trnA(tgc)	6276	6344	-	69		
trnN(gtt)	6347	6419	-	73		
trnC(gca)	6420	6485	-	66		
trnY(gta)	6485	6555	-	71		
cox1	6557	8107	+	1551	GTG	AGG
trnS2(tga)	8099	8173	-	75		
trnD(gtc)	8176	8244	+	69		
cox2	8246	8929	+	684	ATG	TAA
trnK(ttt)	8931	8998	+	68		

atp8	9000	9167	+	168	ATG	TAA
atp6	9158	9841	+	684	ATG	TAA
cox3	9841	10624	+	784	ATG	T(AA)
trnG(tcc)	10625	10693	+	69		
nad3	10694	10866	+	352	ATG	TAA
	10868	11045	+			
trnR(tcg)	11047	11115	+	69		
nad4l	11116	11412	+	297	ATG	TAA
nad4	11406	12783	+	1378	ATG	T(AA)
trnH(gtg)	12784	12852	+	69		
trnS1(gct)	12853	12923	+	70		
trnL1(tag)	12924	12994	+	71		
nad5	12995	14815	+	1821	ATG	TAA
cob	14815	15957	+	1143	ATG	TAA
trnT(tgt)	15961	16030	+	70		
trnP(tgg)	16033	16102	-	70		
nad6	16108	16629	-	522	ATG	AGG
trnE(ttc)	16631	16698	-	68		
