# Supplementary material for: Mitogenomic Insights into the Evolution, Divergence Time, and Ancestral Ranges of Coturnix Quails
Source: Genes (Basel). 2024 Jun 5;15(6):742. doi: 10.3390/genes15060742 (PMC11202683; doi:10.3390/genes15060742)
Supplement: Supplementary file 1 [file genes-15-00742-s001.zip › Table S5.pdf]

Table S5. Nucleotide composition of *C. coturnix* mitogenome.

	Size (bp)	A%	C%	G%	T%	A+T%	G+C%	AT-skew	GC-skew
Mitogenome	16696	31.1	31.2	13.1	24.6	55.7	44.3	0.117	-0.409
PCGs	11399	29.4	32.2	12.7	25.7	55.1	44.9	0.067	-0.434
tRNA	1546	33.1	24.6	17.5	24.8	57.9	42.1	0.143	-0.169
rRNA	2587	34.1	27.0	18.0	20.9	55	45	0.240	-0.200
D-loop	1158	26.5	25.6	13.7	34.2	60.7	39.3	-0.127	-0.303
