# Supplementary material for: Mitogenomic Insights into the Evolution, Divergence Time, and Ancestral Ranges of Coturnix Quails
Source: Genes (Basel). 2024 Jun 5;15(6):742. doi: 10.3390/genes15060742 (PMC11202683; doi:10.3390/genes15060742)
Supplement: Supplementary file 1 [file genes-15-00742-s001.zip › Table S6.pdf]

Table S6. Nucleotide composition of *C. japonica* mitogenome.

	Size (bp)	A%	C%	G%	T%	A+T%	G+C%	AT-skew	GC-skew
Mitogenome	16698	31.1	31.3	13.1	24.5	55.6	44.4	0.119	-0.410
PCGs	11399	29.4	32.2	12.7	25.7	55.1	44.9	0.067	-0.434
tRNA	1477	32.6	25.1	17.8	24.4	57	42.9	0.144	-0.170
rRNA	2589	34.3	27	17.8	20.9	55.2	44.8	0.243	-0.205
D-loop	1155	25.9	26.1	14.2	33.9	59.8	40.3	-0.134	-0.295
