# Supplementary figures and images for: Mitogenomic Insights into the Evolution, Divergence Time, and Ancestral Ranges of Coturnix Quails
Source: Genes (Basel). 2024 Jun 5;15(6):742. doi: 10.3390/genes15060742 (PMC11202683; doi:10.3390/genes15060742)

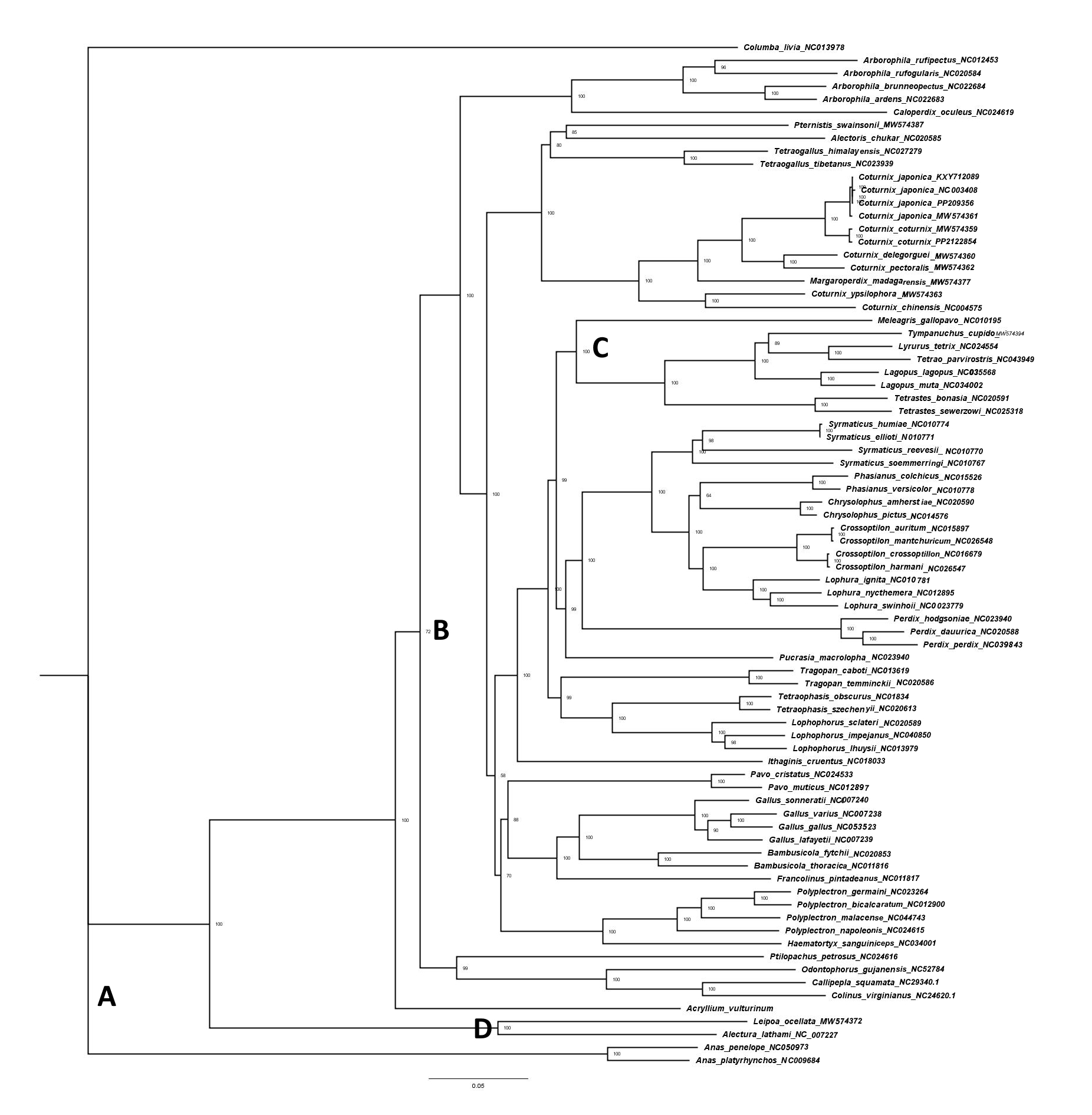

Supplement: Supplementary file 1 [file genes-15-00742-s001.zip › Figure S1.jpg]

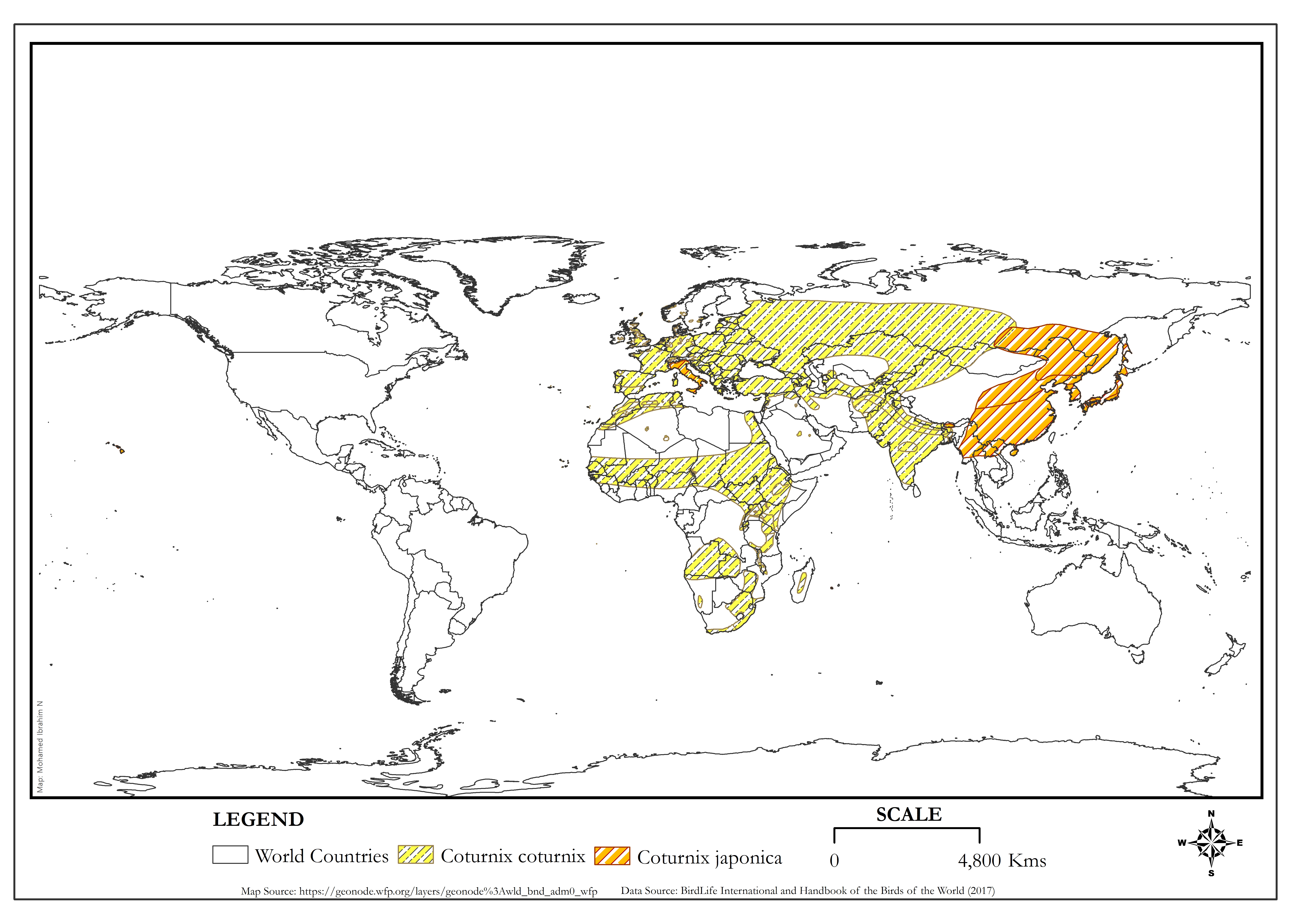

Supplement: Supplementary file 1 [file genes-15-00742-s001.zip › Figure S10.jpg]

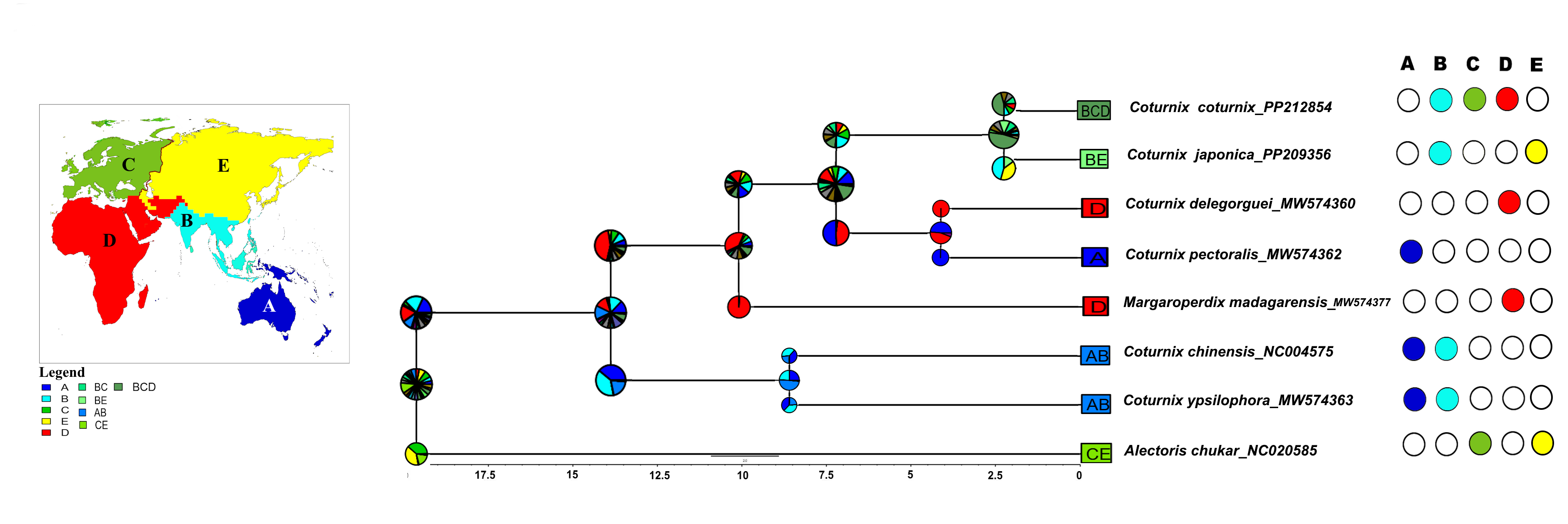

Supplement: Supplementary file 1 [file genes-15-00742-s001.zip › Figure S11.jpg]

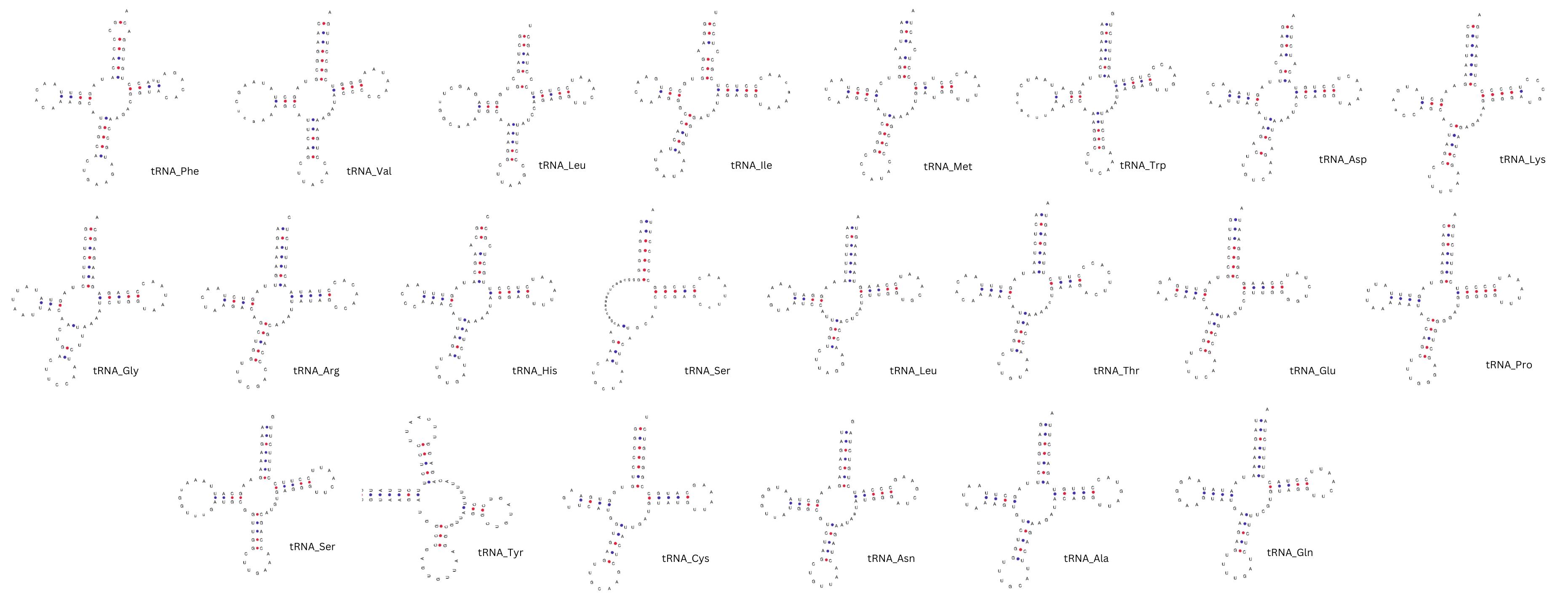

Supplement: Supplementary file 1 [file genes-15-00742-s001.zip › Figure S2.png]

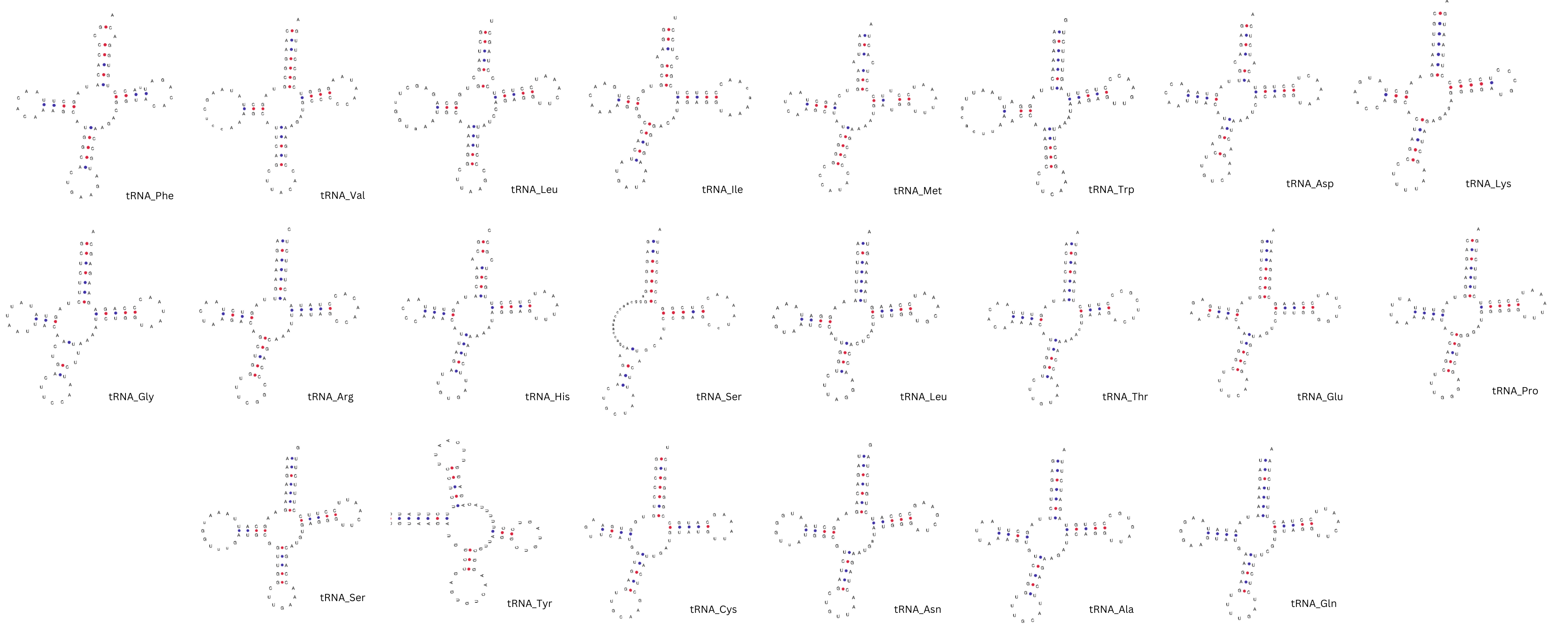

Supplement: Supplementary file 1 [file genes-15-00742-s001.zip › Figure S3.png]

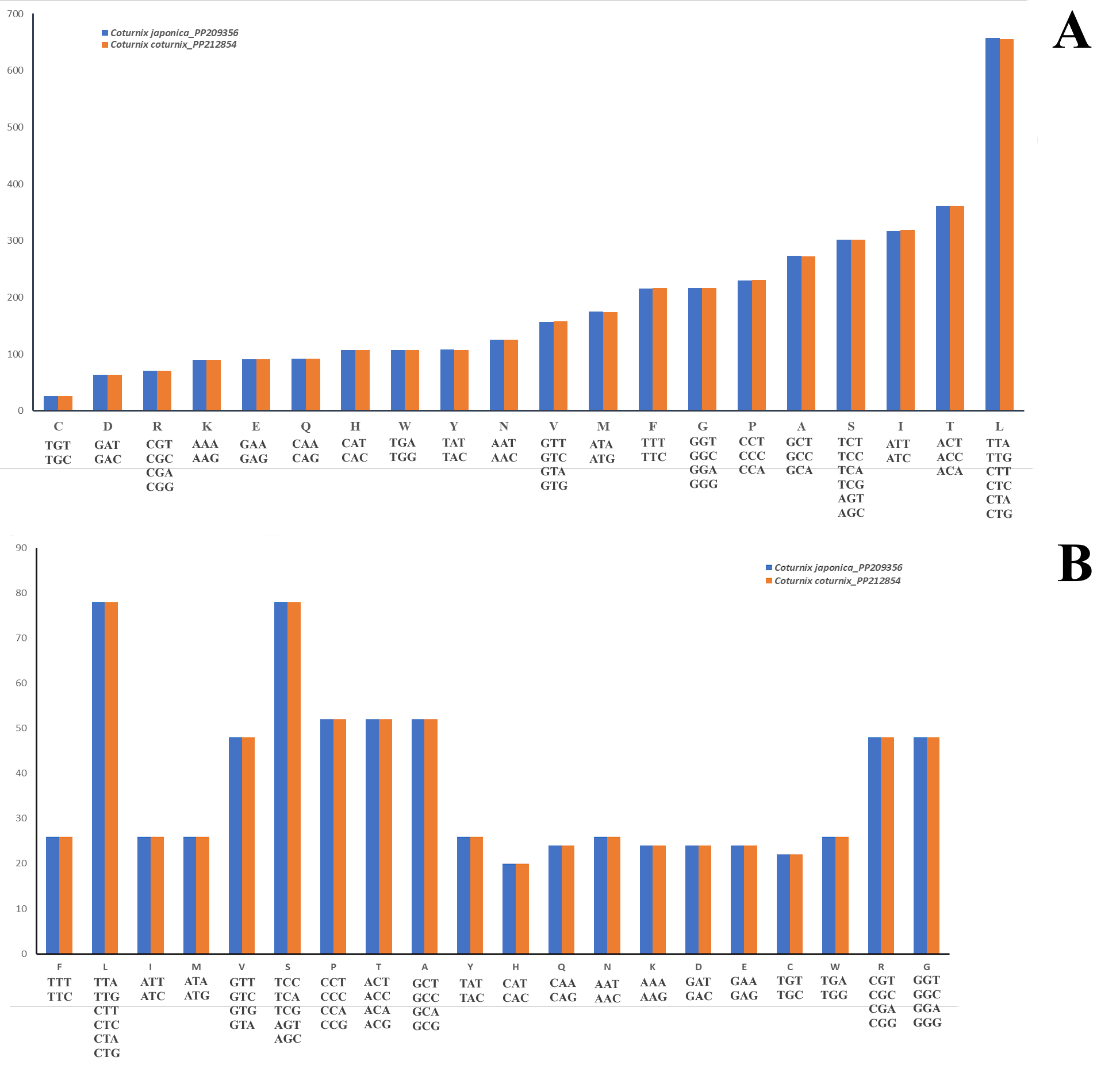

Supplement: Supplementary file 1 [file genes-15-00742-s001.zip › Figure S4.jpg]

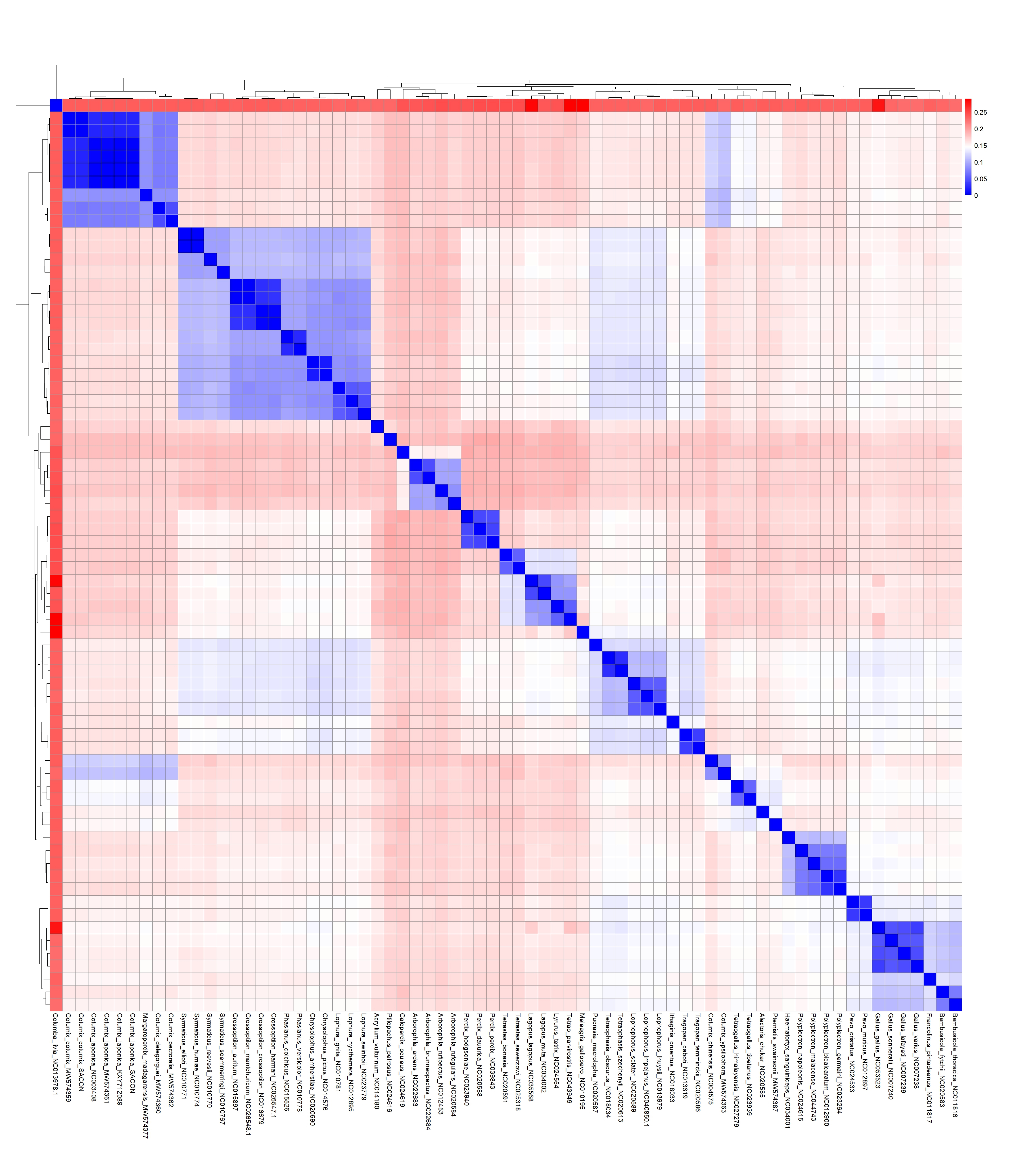

Supplement: Supplementary file 1 [file genes-15-00742-s001.zip › Figure S6.jpg]

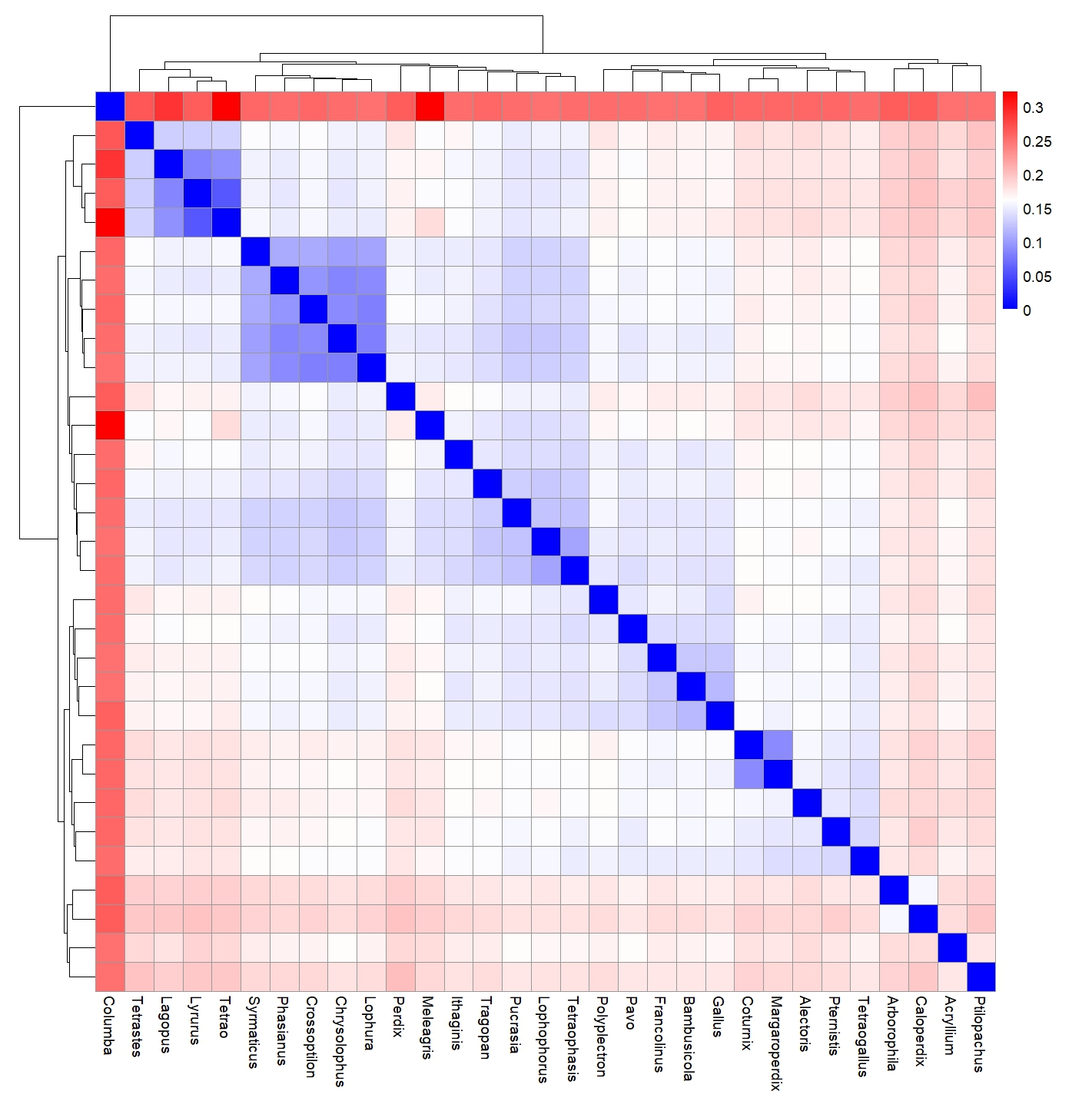

Supplement: Supplementary file 1 [file genes-15-00742-s001.zip › Figure S7.jpg]

Estimates

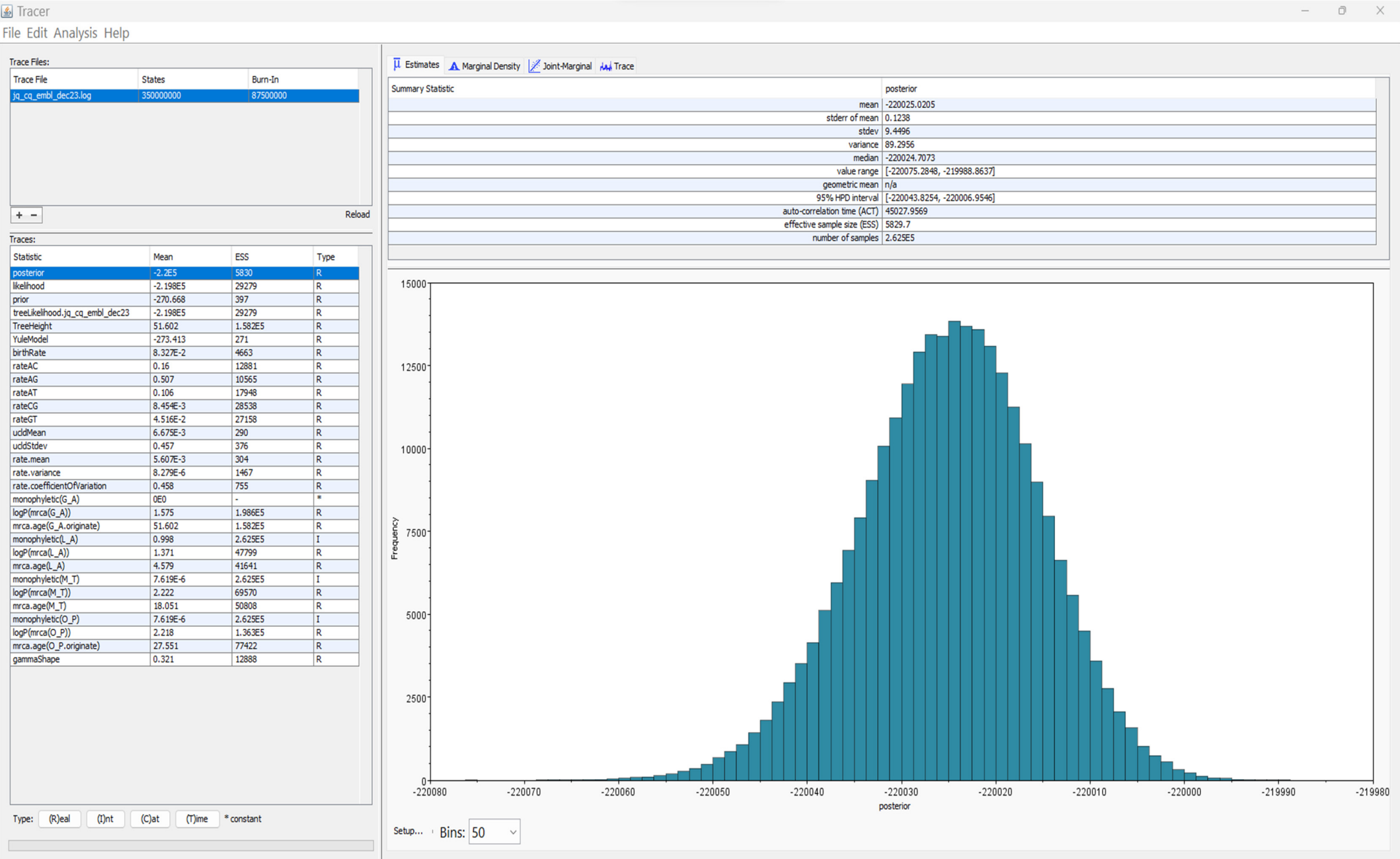

Marginal Density

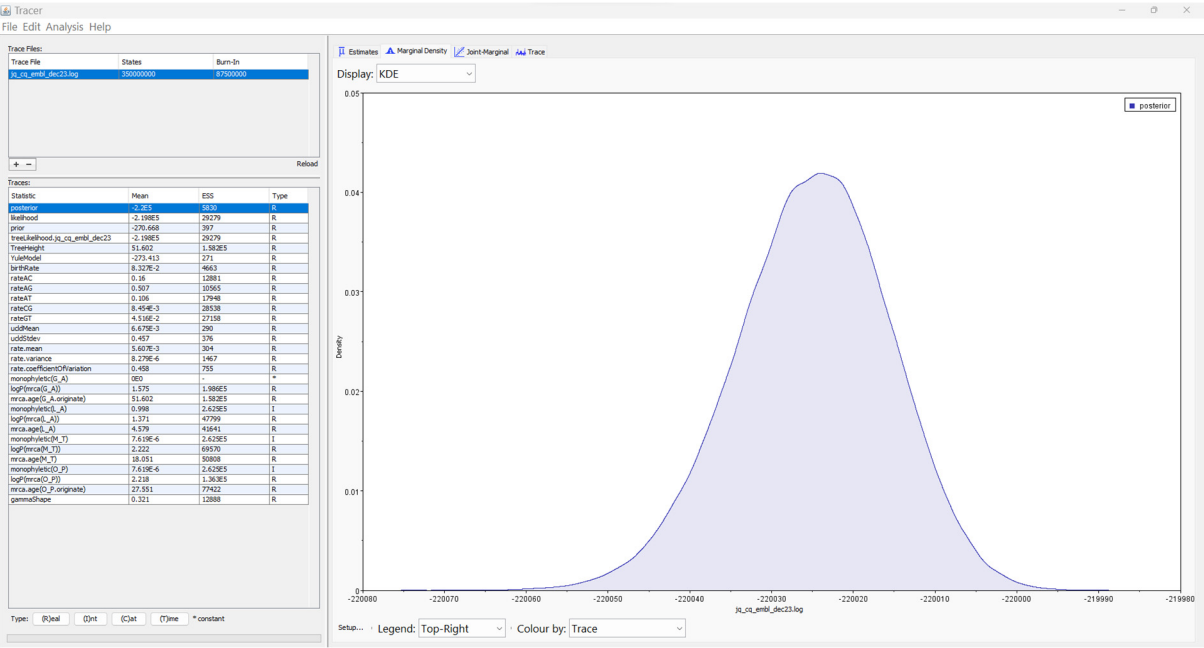

Trace

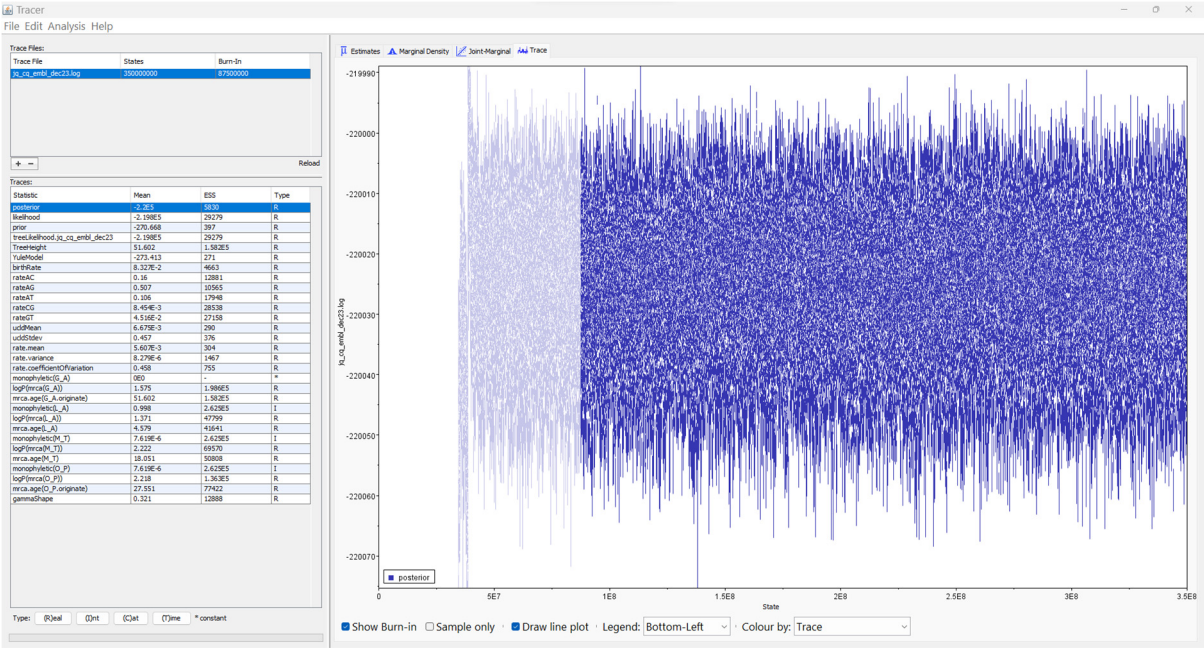

Supplement: Supplementary file 1 [file genes-15-00742-s001.zip › Figure S8.pdf]

Estimates

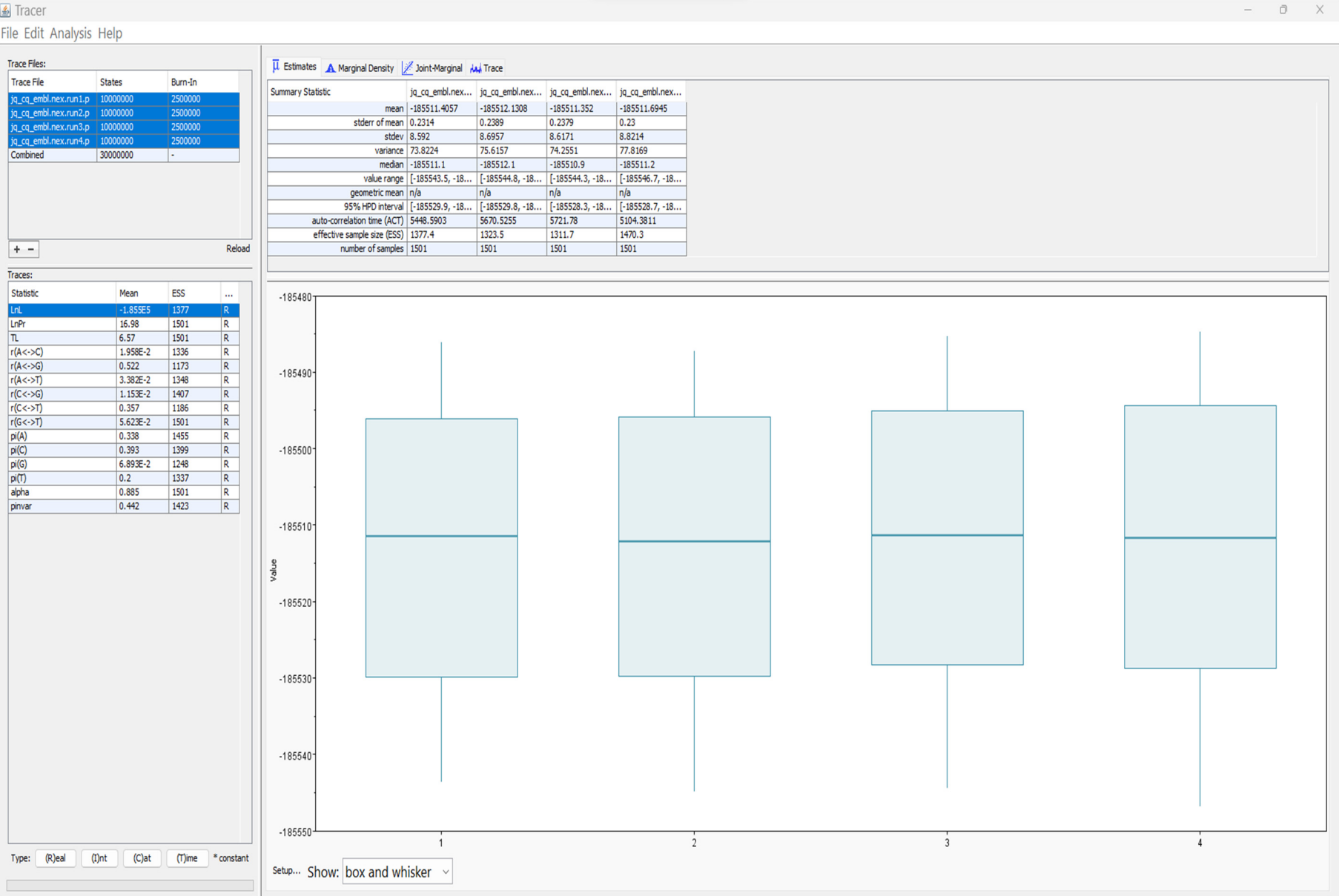

Marginal Density

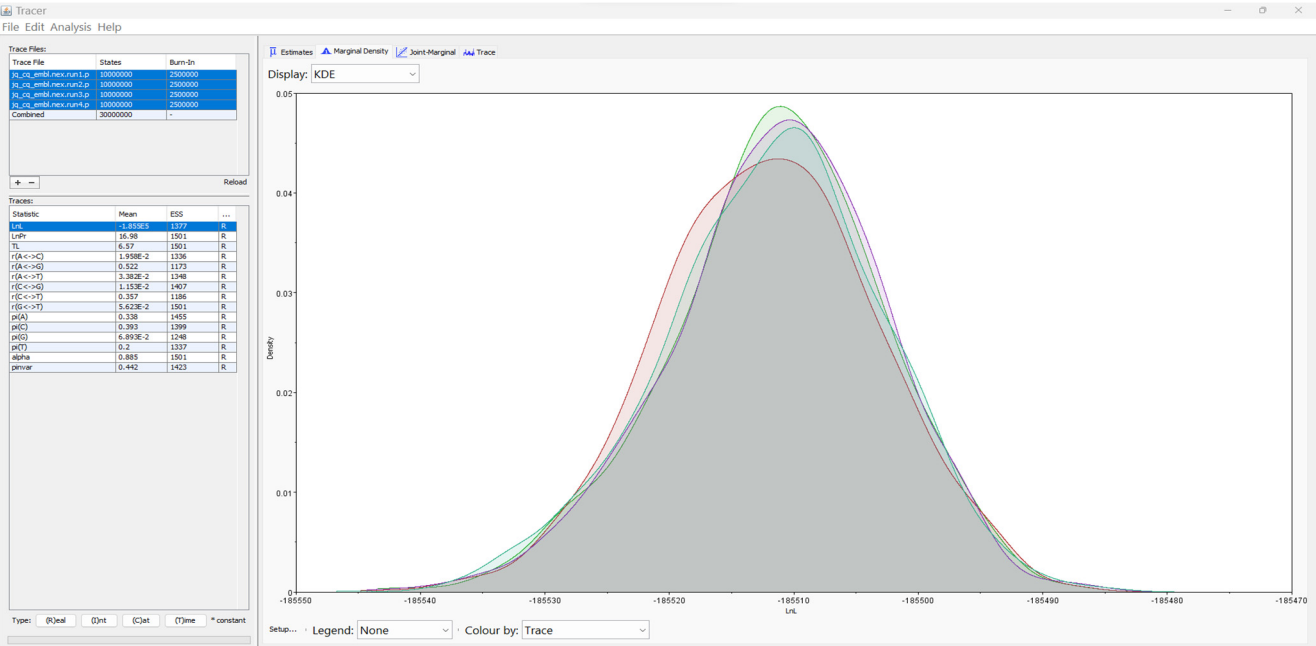

Trace

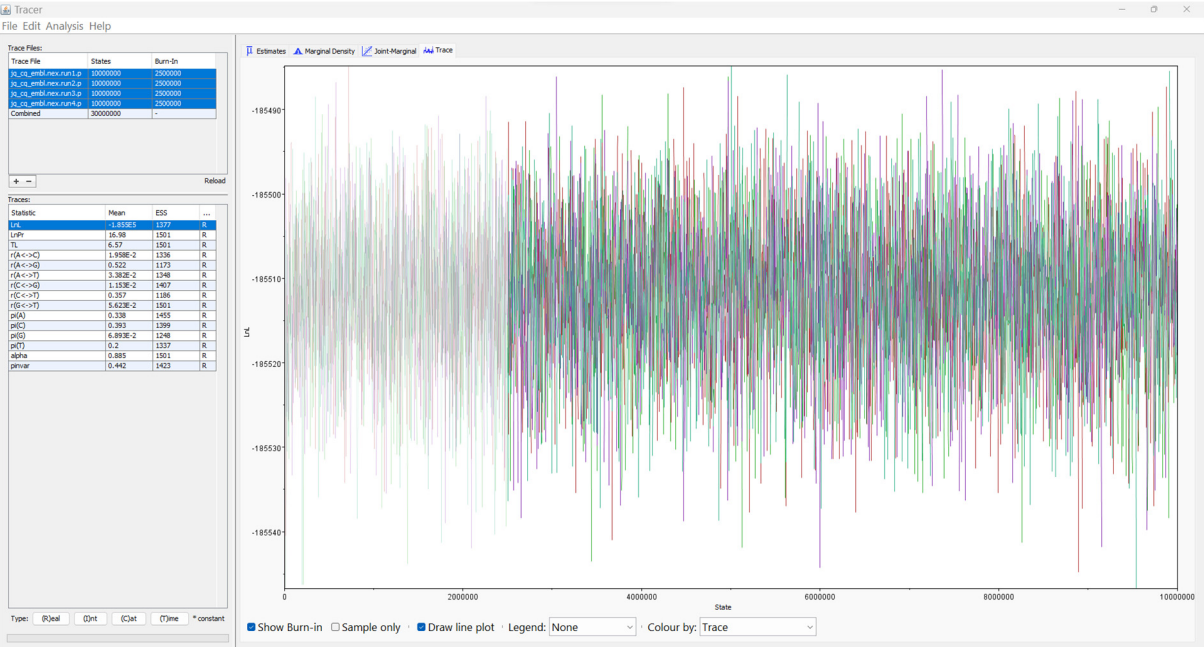

Joint Marginal

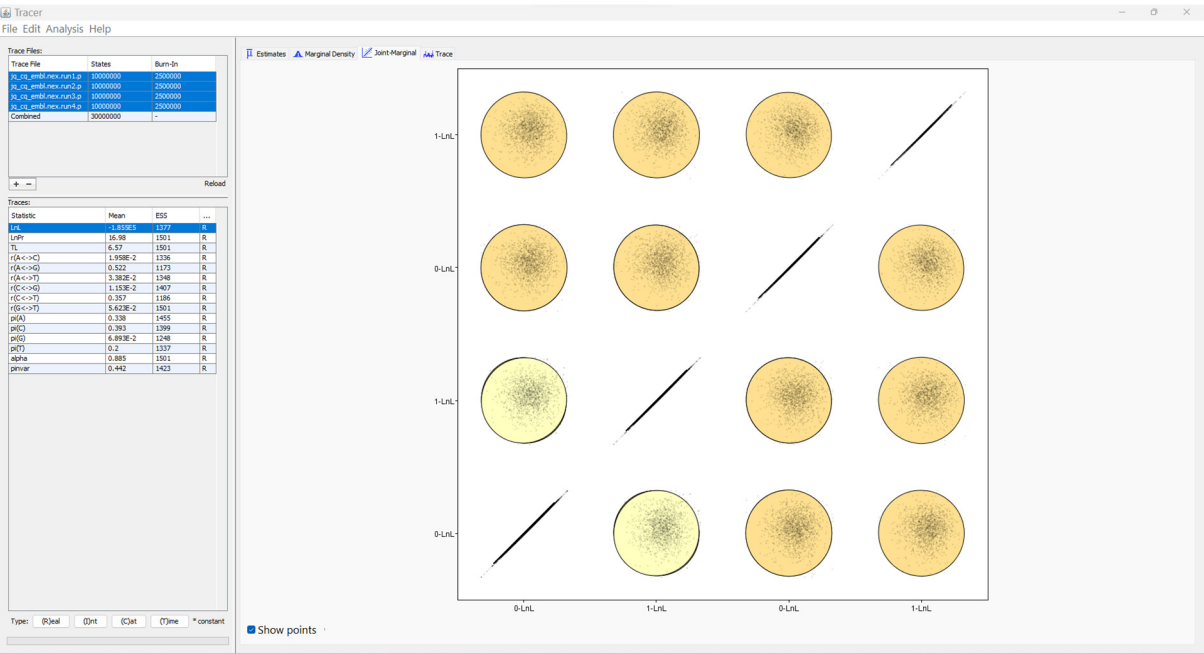

Supplement: Supplementary file 1 [file genes-15-00742-s001.zip › Figure S9.pdf]
